# Membrane-Interacting DNA Nanotubes Induce Cancer Cell Death

**DOI:** 10.3390/nano11082003

**Published:** 2021-08-04

**Authors:** Samet Kocabey, Aslihan Ekim Kocabey, Roger Schneiter, Curzio Rüegg

**Affiliations:** 1 Department of Oncology, Microbiology and Immunology, Faculty of Science and Medicine, University of Fribourg, Chemin du Musée 18, PER17, 1700 Fribourg, Switzerland; 2 Department of Biology, Faculty of Science and Medicine, University of Fribourg, Chemin du Musée 10, PER05, 1700 Fribourg, Switzerland; aslihan.ekimkocabey@unifr.ch (A.E.K.); roger.schneiter@unifr.ch (R.S.)

**Keywords:** DNA nanotechnology, DNA nanostructure, targeted delivery, cytochrome c, cholesterol, cytotoxicity

## Abstract

DNA nanotechnology offers to build nanoscale structures with defined chemistries to precisely position biomolecules or drugs for selective cell targeting and drug delivery. Owing to the negatively charged nature of DNA, for delivery purposes, DNA is frequently conjugated with hydrophobic moieties, positively charged polymers/peptides and cell surface receptor-recognizing molecules or antibodies. Here, we designed and assembled cholesterol-modified DNA nanotubes to interact with cancer cells and conjugated them with cytochrome c to induce cancer cell apoptosis. By flow cytometry and confocal microscopy, we observed that DNA nanotubes efficiently bound to the plasma membrane as a function of the number of conjugated cholesterol moieties. The complex was taken up by the cells and localized to the endosomal compartment. Cholesterol-modified DNA nanotubes, but not unmodified ones, increased membrane permeability, caspase activation and cell death. Irreversible inhibition of caspase activity with a caspase inhibitor, however, only partially prevented cell death. Cytochrome c-conjugated DNA nanotubes were also efficiently taken up but did not increase the rate of cell death. These results demonstrate that cholesterol-modified DNA nanotubes induce cancer cell death associated with increased cell membrane permeability and are only partially dependent on caspase activity, consistent with a combined form of apoptotic and necrotic cell death. DNA nanotubes may be further developed as primary cytotoxic agents, or drug delivery vehicles, through cholesterol-mediated cellular membrane interactions and uptake.

## 1. Introduction

DNA nanostructures have been widely used to understand biological processes and to improve diagnostic and therapeutic repertoires. DNA self-assembly allows for the generation of specifically shaped objects at the nanoscale with highly predictable structures that can be further functionalized by conjugation with other biomolecules [1]. This flexibility and programmability of DNA nanostructures makes it possible to mimic the function of complex biological elements, including membrane-associated proteins [2,3] and pore complexes [4]. Moreover, these structures could be used as nanoscale platforms to precisely arrange the position of ligands, induce cancer specific cell signaling pathways and thereby regulate cancer cell behavior. For example, ephrin-A5 ligand functionalized nanocalipers were used to decrease the invasiveness of breast cancer cells [5]. In another study, DNA origami structures decorated with both integrin specific peptides and epidermal growth factor (EGF) ligands were utilized in the investigation of cancer cell adhesion. These structures increased cell spreading when the ligands were positioned 60 nm apart [6]. In addition, recent studies have demonstrated the enhancement of apoptosis signaling through a hexagonal arrangement of the death receptor Fas ligands (FasL) or TRAIL (tumor necrosis factor-related apoptosis-inducing ligand) mimicking peptides on designed DNA structures [7,8]. When DNA origami structures were decorated with specific ligands, T-cells were activated [9], and tumor cells could be killed by CD8^+^ cytotoxic T cells [10]. In addition, to modulate cellular behavior, DNA origami structures have also been used as nanocarriers for delivery purposes, by loading them with antibody fragments [11], enzymes [12] or cytotoxic drugs [13,14]. For the intracellular delivery of these molecules and to increase the stability of the structures in biological media, DNA nanostructures are modified with either specific molecules/ligands [15,16,17], antibodies [18] or aptamers [19,20] that interact with membrane receptors, such as folate, transferrin and nucleolin, respectively, or with cationic polymers [21], peptoids [22], oligolysine-based positively charged peptides [23] or hydrophobic lipid molecules [24].

Among these approaches, modification of DNA with lipid molecules may have potential advantages due to the ability to conjugate a broad range of lipids and thereby increase their targeting to the cell surface/plasma membrane. Lipid-modified DNA nanostructures have been extensively studied on artificial lipid membranes as biomimetic nanopores to understand and regulate the transport of ions [25,26], charged fluorescent dyes [27], cytotoxic drugs [28] and the sensing of analytes [29]. DNA origami structures modified with lipid moieties were also used to mimic membrane-assisted assembly of proteins, to induce membrane curvature [2,30] and to study membrane fusion processes [31]. In addition to these in vitro applications, lipid modified DNA nanostructures have been recently used in vivo to reveal cellular interactions. These lipid-modified DNA nanostructures bind to cancer cell membranes [32], white blood cells [33] or endothelial cells [34]. Lipid-modified DNA nanostructures can also be used as membrane-spanning nanopores for doxorubicin transport [35] or to encapsulate DNA origami nanostructures within liposomes to improve their in vivo stability [24].

Protein-based anticancer therapy is one of the most efficient strategies for cancer treatment due to specific targeting, resulting in improved efficacy and reduced side effects compared to classical chemotherapeutic agents. Proteins can be conjugated efficiently to DNA-based delivery systems owing to their large surface size and multiple attachment sides. Cytochrome c is one such potential protein that could be used as an anticancer drug. It is a small (12 kDa), highly conserved protein that plays a key role in the mitochondrial electron transport chain. In response to external (e.g., death receptor activation, TRAIL and Fas) or internal (e.g., DNA damage) signals, cytochrome c is released from the mitochondria to the cytoplasm through Bax/Bid-mediated outer mitochondrial membrane permeabilization [36]. Cytoplasmic cytochrome c induces apoptosis by interacting with Apaf-1 to form a large molecular complex, activating caspase 9. In turn, this activates caspase 3, leading to cell death [37,38]. In many cancer types, however, the mitochondrial release mechanism of cytochrome c is reduced due to mutations in upstream signaling or Bcl2 overexpression, resulting in diminished apoptosis. To overcome this limitation, direct delivery of cytochrome c to the cytoplasm of cancer cells may be considered [39,40]. However, like most proteins, cytochrome c, is membrane-impermeable. Thus, formulations for its intracellular delivery need to be devised [41]. Previously, several groups have shown that the cytoplasmic delivery of cytochrome c can be achieved either via conjugation to ligands of cell surface receptors, such as transferrin and folate, or by its encapsulation in polymers [42,43,44].

In this study, we designed and synthesized cholesterol-modified DNA nanotubes conjugated with cytochrome c and investigated their interaction with cancer cells and intracellular localization of the delivered cytochrome c. We demonstrate that cholesterol-modified DNA nanotubes, but not unmodified ones, effectively bound to the cell surface, were taken up via endocytosis and induced cell death. Cytochrome c conjugated to these cholesterol-modified DNA nanotubes did not interfere with their surface binding and uptake and did not further enhance cell death.

## 2. Materials and Methods

### 2.1. DNA Nanotube Design

DNA nanotubes were designed via the single-stranded tile (SST) method as shown previously [45] by mixing 9 different oligonucleotides (ODN) consisting of 48 or 84 bases. These include four unmodified oligonucleotides, three cholesterol modified oligonucleotides (LubioScience-Switzerland IDT, Zurich, Switzerland), azide-modified oligonucleotide for click reactions (Biomers, Ulm, Germany) and Alexa Fluor 647-labelled oligonucleotide for microscopy (Eurofins, Ebersberg, Germany) (Appendix A). All modified oligonucleotides were purified using HPLC. The sequences of the DNA strands are given in Table A1.

### 2.2. Cytochrome c Conjugation

Cytochrome c (Sigma Aldrich, Buchs, Switzerland) was conjugated to 5′ azide-modified oligonucleotides through cysteines using a dibenzocyclooctine (DBCO)-maleimide linker-based click reaction [46]. First, 1–4 mg cytochrome c was dissolved in 1 mL Tris-HCl buffer (100 mM, pH 7.2). Tris(2-carboxyethyl)phosphine (TCEP) solution (50 mM, Sigma Aldrich, Buchs, Switzerland) was then added at 1:10 (*v*/*v*) to the 100 µL cytochrome c solution, and the mixture was incubated for 2 h at RT (400 rpm). Excess TCEP was removed by buffer exchange using 3K Amicon Ultra 0.5 mL centrifuge filters (Merck Millipore, Darmstadt, Germany) by spinning four times for 6 min at 14,000× *g* at 4 °C. After each round, the flow through was discarded, and the centrifuge filter tube was filled again with buffer to 500 µL. The protein concentration was estimated by measuring absorbance at 280 nm using a Nanodrop (Thermo Fisher Scientific, Basel, Switzerland). Then, reduced cytochrome c (10 nmol) was mixed with a 50-fold excess of DBCO-maleimide (Sigma Aldrich, Buchs, Switzerland, 50 mM in dimethylformamide (DMF)), and the reaction was incubated at 4 °C on a shaker in the dark overnight. The next day, excess DBCO-maleimide was removed again using Amicon 3K filters, and the DBCO-conjugated cytochrome c was mixed with azide-modified oligonucleotides (1:2) for 1 h at RT on a shaker (400 rpm). The oligonucleotide-conjugated cytochrome c was analyzed by running samples on 12% sodium dodecyl sulfate-polyacrylamide gel electrophoresis (SDS-PAGE). Proteins were stained either with Coomassie blue (Sigma Aldrich, Buchs, Switzerland) or No-Stain protein-labeling reagent (Thermo Fisher Scientific, Basel, Switzerland).

To determine if cytochrome c conjugates possessed peroxidase activity, 100 µL aliquots of 3,3′,5,5′-Tetramethylbenzidine (TMB) solution (Sigma Aldrich, Buchs, Switzerland) were dispensed in a 96-well plate. Subsequently, 5 µL of cytochrome c or cytochrome c-ODN conjugate were added to the corresponding wells, followed by the addition of 20 µL of H_2_O_2_. The absorbance was measured at 655 nm with a spectrophotometry (TECAN infinite M200PRO, Männedorf, Switzerland).

To fluorescently label cytochrome c, 2 mg/mL Atto488 NHS (*N*-hydroxysuccinimide)-ester (Sigma Aldrich, Buchs, Switzerland) was dissolved in DMF and mixed with 2 mg/mL cytochrome c (0.1 M bicarbonate buffer, pH 8.3) (2:1). Then, the solution was incubated for 1 h at RT on a shaker (400 rpm). Excess Atto488 NHS-ester was removed by 3K Amicon filters.

### 2.3. DNA Nanotube Assembly and Gel Analysis

DNA nanotubes were assembled by mixing all oligonucleotides at equivalent molar ratios at a final concentration of 100 nM to 1 µM in the folding buffer (10 mM Tris-HCl, 1 mM EDTA, 16 mM MgCl_2_, pH 8). The structures were folded over the course of different incubations with increasing incubation times (15 min, 2 h and 16 h) by linear cooling from 65 °C to 25 °C using a Biometra TAdvanced Twin PCR thermocycler (Analytik Jena, Jena, Germany). Assembled DNA nanotubes were then purified using 100K Amicon Ultra 0.5 mL centrifuge filters to remove excess ssDNA that was not folded into the structures. After washing four times, the remaining solution containing DNA nanotubes were collected, and the DNA concentration was determined by measuring the optical density at 260 nm.

DNA nanotubes were analyzed by running samples (10 µL of purified DNA nanotube, 100 nM) in a 2% agarose gel with or without GelGreen dye (0.5× TBE buffer, 11 mM MgCl_2_) using GeneRuler DNA Ladder mix (Thermo Fischer Scientific, Basel, Switzerland) as molecular weight ladder. The gel was run for 2 h at 70 V in an ice-cold water bath to prevent heat-induced denaturation of the DNA nanotubes. Fluorescently labelled nanotubes were visualized in a G-BOX imaging system (Syngene, Synoptic Ltd, Cambridge, UK).

### 2.4. Cell Culture

HeLa cells were purchased from American Type Culture Collection (ATCC, Rockville, MD, USA). Cells were cultured at 37 °C, 5% CO_2_ and 95% humidity in Dulbecco’s modified Eagle’s medium (DMEM) supplemented with Glutamax, 10% fetal bovine serum (FBS) and 1% Penicillin and Streptomycin. Cells were treated with DNA nanotubes in Opti-MEM (Minimal Essential Medium) serum-reduced medium. All cell culture reagents were purchased from Thermo Fischer Scientific (Basel, Switzerland).

### 2.5. Flow Cytometry and Confocal Microscopy

The interaction of the DNA nanotubes with cells was analyzed by flow cytometry using a MACSQuant Analyzer 10 flow cytometer (Miltenyi Biotec, Bergisch Gladbach, Germany), and the data were analyzed with FlowJo Software (*FlowJo*, version 10.6.2; FlowJo LLC: Ashland, OR, USA, 2020). Alexa Fluor 647 intensity was depicted as mean fluorescence intensity (MFI) for all samples. Briefly, cells were precultured for 24 h by seeding onto 24-well plates with a density of 20,000 cells/well. After overnight incubation, the medium was discarded, cells were washed with 1× phosphate buffered saline (PBS) and then treated in triplicates with plain or cholesterol-modified DNA nanotubes (100 nM in Opti-MEM medium) for different time intervals (30 min, 90 min and 3 h). Then, the medium containing DNA nanotubes was aspirated, cells were washed 3 times with 1× PBS, trypsinized and resuspended in FACS buffer (1× PBS, 3% FBS), and transferred into v-bottom 96-well plates (Thermo Fisher Scientific, Basel, Switzerland) for flow cytometry analysis. Viable cells were consistently selected based on forward and side scattering.

For confocal imaging, cells were seeded into Ibidi 18-well polymer coverslip slides with a density of 1000 cells/well and treated with plain or cholesterol-modified nanotubes (100 nM) for up to 3 h in Opti-MEM medium. At the predetermined time points, cells were washed 3 times with 1× PBS, and the medium was replaced with DMEM. Confocal imaging was performed on a Leica TCS SP5 inverted microscope (Leica Microsystems GmbH, Mannheim, Germany) using a Plan-Apochromat 20×/0.7 NA dry objective (Zeiss GmbH, Jena, Germany). Laser lines 405, 488 and 633 were used for Hoechst 33452, Alexa Fluor 647 and Atto488 excitation, respectively. After acquisition, the images were analyzed by Fiji software (*ImageJ*, National Institute of Health, Bethesda, MD, USA).

To visualize the localization of DNA nanotubes, cells were treated with DNA nanotubes as mentioned above. The medium was discarded at the predetermined time points (1 h and 3 h), washed with 1× PBS and incubated with Opti-MEM solution containing both 10 nM BioTracker Orange 560 lysosome dye (Sigma Aldrich, Buchs, Switzerland) and 1µg/mL Hoechst 33342 (Thermo Fisher Scientific, Basel, Switzerland). Cells were washed after 30 min of incubation, and images were collected using a Zeiss LSM 710 inverted confocal microscope (Axio Observer.Z1, Zeiss, Feldbach, Switzerland). Image acquisition was performed using an EC Plan-Neofluar 40×/1.30 NA-immersion oil objective (Zeiss GmbH, Jena, Germany). Laser lines 405, 488 and 514 were used for Hoechst 33452, Atto488 and Biotracker Orange 560 excitation, with the appropriate band-pass filters. Colocalization analysis was performed using the fluorescent intensity profile function of ZEN software (*ZEN*, version 3.3, blue edition; Zeiss GmbH, Jena, Germany).

### 2.6. Apoptosis Assay and Western Blotting

Cellular apoptosis was determined with the Annexin V/ Propidium Iodide Apoptosis assay kit (Thermo Fisher Scientific, Basel, Switzerland) by following the manufacturer’s instructions. Briefly, cells were seeded into 6-well plates with a density of 100,000 cells/well and preincubated overnight in DMEM medium. The next day, the medium was aspirated, and cells were treated with DNA nanotubes (1 µM) either in Opti-MEM or DMEM (10% FBS) media for 4 h or 24 h. As apoptosis and necrosis controls, cells were treated with either 1 µM staurosporine (Abcam, Cambridge, UK) or 3 mM H_2_O_2_, respectively. Then, cells were washed with 1× PBS, trypsinized and transferred into v-bottom 96-well plates. Cells were resuspended in 100 µL of 1× Annexin-binding buffer containing 5 µL of Annexin V-APC and 1 µL of 100 µg/mL PI and incubated for 15 min at RT. Then, 100 µL of 1× Annexin-binding buffer was added to the cell suspension and mixed gently. Cells were analyzed by flow cytometry using the MACSQuant Analyzer 10 flow cytometer (Miltenyi Biotec, Bergisch Gladbach, Germany). For data analysis, FlowJo Software (v10.6.2, FlowJo LLC) was utilized.

In order to inhibit caspase activity, cells were treated with the pan caspase inhibitor, Z-VAD-FMK (carbobenzoxy-valyl-alanyl-aspartyl-[O-methyl]- fluoromethylketone) (400 µM, R&D Systems, Minneapolis, MN, USA) for 1 h. Then, cells were mixed with an equal volume of cell medium containing DNA nanotubes or staurosporine.

For the Western blot analysis, cells were trypsinized after 24 h of incubation with DNA nanotubes (1 µM) and lysed using 1× Radioimmunoprecipitation Assay (RIPA) buffer containing 1× protease inhibitor cocktail and phosphatase inhibitors (1 mM phenylmethylsulfonylfluoride (PMSF), as well as 1 mM sodium orthovanadate (Na_3_VO_4_)) (Sigma Aldrich, Buchs, Switzerland). After vigorous mixing and cooling on ice for 5 min, protein lysates were centrifuged at 13,000 rpm for 15 min at 4 °C and cleared supernatants were collected. Protein concentration was measured using a BC assay (Interchim, Montluçon, France), where bovine serum albumin (BSA) was used as the standard. Proteins were run on a 12% reducing sodium dodecyl sulfate (SDS) polyacrylamide gel and transferred to a nitrocellulose membrane (Amersham, United Kingdom) for 45 min at 130 mA. Membranes were blocked with 5% BSA for at least 30 min and then incubated with primary antibodies (Table A2) at 4 °C overnight. The next day, membranes were washed with 1× tris-buffered saline, 0.1% Tween 20 (TBST) for three times and incubated with the corresponding horseradish peroxidase (HRP)-coupled secondary antibodies for 1 h at room temperature. After the washing steps, protein detection was performed by a short incubation with the enhanced chemiluminescence (ECL) reagent (Sigma Aldrich, Buchs, Switzerland). Signals were detected by ImageQuant Las 4000 (GE Healthcare Life Sciences, Marlborough, MA, United States). The band intensities were calculated using ImageJ software. The relative expression of proteins was normalized to the expression of β-actin.

### 2.7. MTT Cell Viability Assay

HeLa cells were seeded in 96-well plates with a density of 10,000 cells/well and preincubated overnight in DMEM medium. The next day, the medium was aspirated, and cells were treated with DNA nanotubes at different concentrations (100 nM, 250 nM, 500 nM and 1µM) in Opti-MEM for 24 h. Then, the medium was discarded, and cells were incubated in 100 µL of fresh medium containing 0.5 mg/mL MTT (3-(4,5-dimethythiazol-2-yl)-2,5-diphenyl tetrazolium bromide, Sigma Aldrich, Buchs, Switzerland) for 2 h. After incubation, the medium was discarded again, the MTT formazan product was solubilized with 100 µL dimethyl sulfoxide (DMSO). The absorbance was measured at 570 nm with a spectrophotometry (TECAN infinite M200PRO, Männedorf, Switzerland).

### 2.8. Dye Efflux Analysis by Flow Cytometry

HeLa cells were seeded in 24-well plates with a density of 100,000 cells/well and preincubated overnight in DMEM medium. The next day, the medium was aspirated, and cells were incubated in 500 µL of fresh medium containing 10 µM Atto488 NHS-ester (Sigma Aldrich, Buchs, Switzerland) for 2 h. Then, cells were washed with 1× PBS for several times and treated with DNA nanotubes or the apoptosis inducer, staurosporine, at different concentrations (100 nM, 250 nM, 500 nM and 1µM) for 3 h. Cells were analyzed by flow cytometry using the MACSQuant Analyzer 10 flow cytometer (Miltenyi Biotec, Bergisch Gladbach, Germany). For data analysis, FlowJo Software (v10.6.2, FlowJo LLC) was utilized.

## 3. Results

### 3.1. Design and Self-Assembly of DNA Nanotubes

**Scheme 1:**
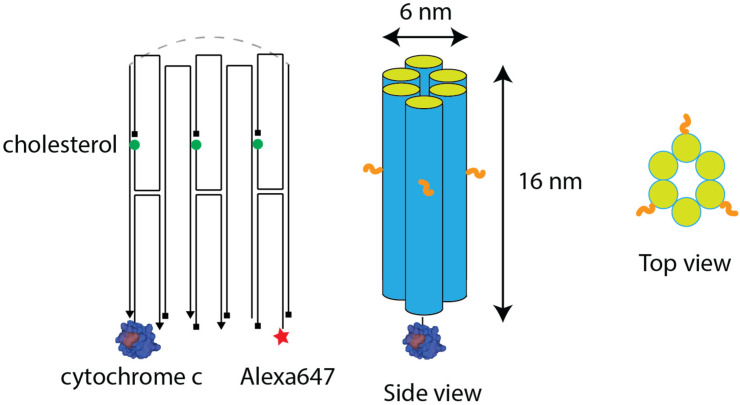
Design of amphiphilic 6-helix DNA nanotubes. Depiction of DNA nanotubes in 2D and 3D views. DNA tile strands are marked with arrows (3′ end) and squares (5′ ends). The length of the tiles was 48 and 84 bases, respectively. Cholesterol molecules are represented as green circles. Cytochrome c and Alexa Fluor 647 fluorophore were conjugated to the 5′ and 3′ ends of two tiles, respectively, as indicated.

Our six-helix DNA nanotubes (6HT) consisting of nine oligonucleotides were designed using a single-stranded tile-based assembly method by modifying previously used sequences as described in Kocabey et al. [16]. In this design, 3 of the oligonucleotides are 84 bases long and 6 of the oligonucleotides are 48 bases long. This design allows for longer complementation of single-tile segments with neighboring tiles (21 base pairings) in the assembly, and hence provides higher thermal stability and resistance to Mg^2+^ depletion as demonstrated previously [16]. Another advantage of this design is its simplicity in terms of minimal preparation time and cost-effective assembly (see Table A1). To promote the interaction of DNA nanotubes with cellular membranes, up to three of the oligonucleotides were tagged from their 3′ ends with cholesterol using tetraethylene glycol (TEG) linkers, seven bases apart from the double crossovers that are located at the center of the structure. To monitor the cellular interaction and the internalization of the nanotubes, one of the oligonucleotides was labelled with the Alexa Fluor 647 dye at its 3′ end. For conjugation with cytochrome c, an oligonucleotide with an azide modification was used, allowing for a click reaction. The length of the designed DNA nanotubes was ~16 nm with an expected diameter of ~6 nm (Scheme 1). It is important to note that the length of the nanotube was longer than the thickness of the cellular lipid bilayer (~5–6 nm), which allowed it to span the lipid bilayer throughout its hydrophobic core.

**Figure 1:**
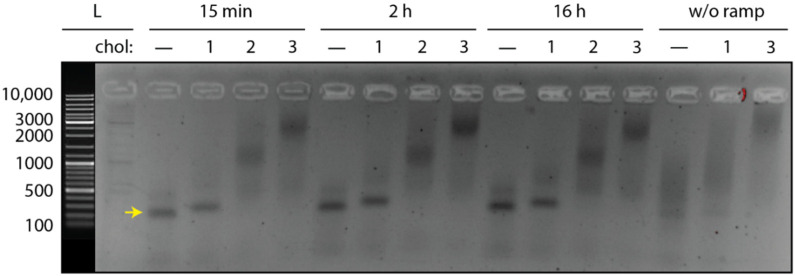
Characterization of amphiphilic 6-helix DNA nanotubes. Agarose gel analysis of DNA nanotubes self-assembled at different annealing times with varying numbers of cholesterol (from 0 to 3 cholesterol per nanotube). L, molecular weight marker (base pair length).

DNA oligonucleotides were assembled into nanotube structures by mixing cholesterol-modified oligonucleotides and unmodified oligonucleotides in Mg^+2^-containing buffer (1× TE 16 mM Mg^+2^) using a thermal annealing process starting at 65 °C and cooling down to room temperature over the course of different time intervals (15 min, 2 h and 16 h). Agarose gel analysis revealed that the nanotubes were assembled properly already after 15 min of thermal annealing (Figure 1). The prominent bands representing the folded structures (252 base pairs (bp) + 36 bases, highlighted by arrow) were apparent between the 200 bp and 300 bp marker bands for the nanotubes without cholesterol at all time points. Increasing the amount of cholesterol anchors led to the formation of hetero-dimers (1× cholesterol) and aggregates (2× and 3× cholesterols) due to hydrophobic interactions between the cholesterol moieties in the aqueous solution. The mobility of the bands in the gel decreased accordingly. Cholesterol molecules tend to form aggregates when their concentration exceeds the critical micelle concentration of cholesterol in solution, 25–40 nM. [47] This is lower than the concentration of our cholesterol-modified DNA nanotubes (>100 nM). However, when the mixtures of DNA strands were kept at room temperature, there were no sharp bands representing the nanotubes. Instead, smearing of bands were observed, indicating that the structures were not properly assembled in the absence of thermal annealing (Figure 1).

### 3.2. Cytochrome c Conjugation and Integration into the DNA Nanotubes

**Figure 2:**
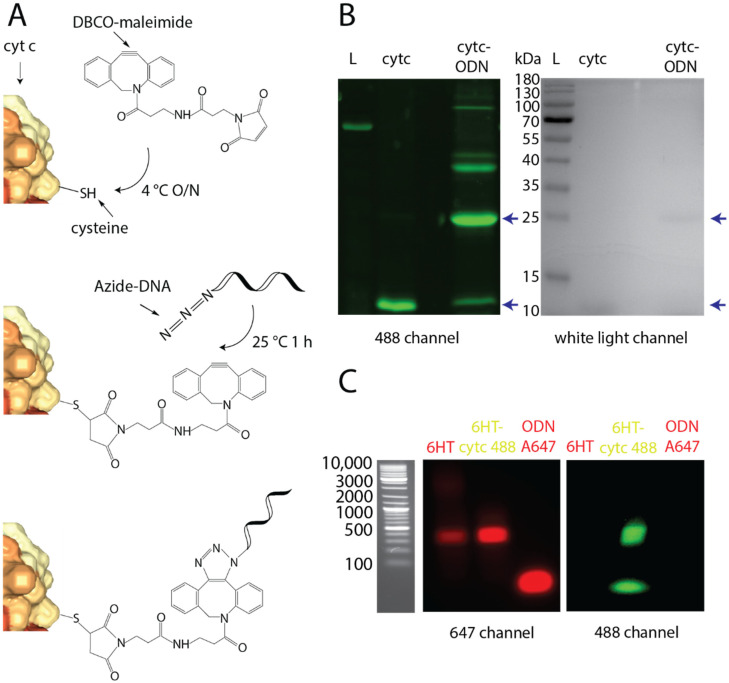
Conjugation of ssDNA to cytochrome c and gel characterization of adducts. (**A**) Stepwise conjugation of azide-modified ssDNA to cytochrome c using a DBCO-maleimide based click reaction. (**B**) SDS-PAGE analysis of DNA-protein conjugates. Cytochrome c and ssDNA-conjugated cytochrome c were stained with the fluorescent No-Stain labeling reagent, visualized in both the 488 nm and white-light channels and highlighted with arrows. (**C**) Agarose gel analysis of DNA nanotube assembly after mixing and annealing of Atto488-labelled cytochrome c-conjugated ssDNA with unconjugated tiles. Gels were visualized in both the 647 nm and 488 nm channels to distinguish between the 6-helix DNA nanotubes (6HT) with or without cytochrome c conjugation. L, molecular weight marker.

**Figure A1:**
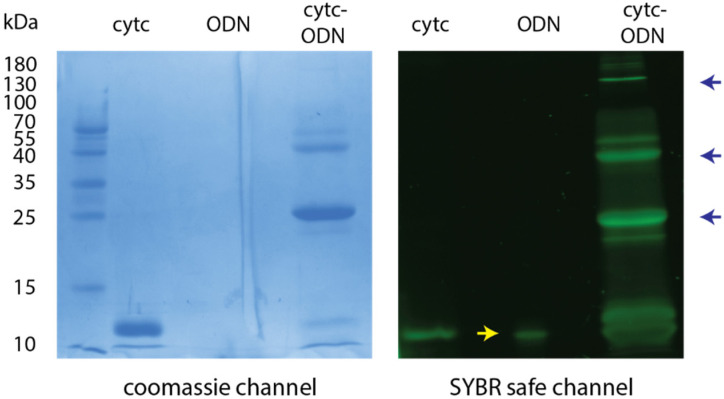
SDS-PAGE analysis of the DNA-protein conjugates. Samples were run in 12% PAGE gel under reducing conditions and stained with Coomassie blue for cytochrome c detection and SYBR safe for DNA detection. Oligonucleotide-conjugated cytochrome c was seen in both Coomassie and SYBR safe stainings, whereas oligonucleotides were detected by SYBR safe staining only (highlighted by arrows).

**Figure A2:**
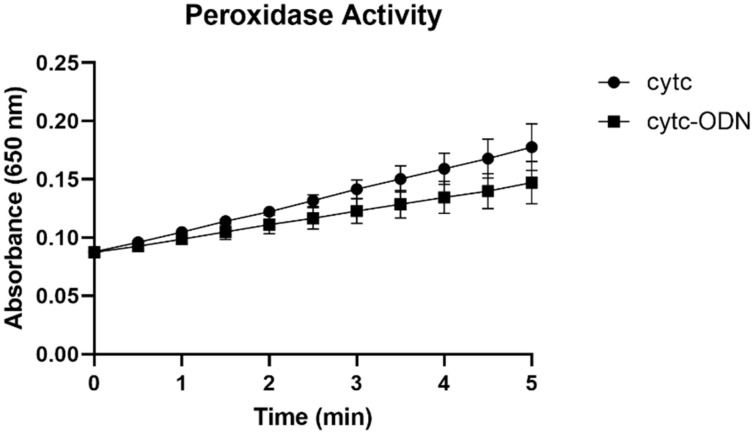
Peroxidase activity of cytochrome c and cytochrome c-conjugated ODN. Kinetic analysis of colorimetric TMB substrate oxidation upon incubation with cytochrome c and cytochrome c-conjugated ODN in the presence of 0.03% H_2_O_2_.

Cytochrome c is highly conserved across species, with a size of 12 kDa. Yeast cytochrome c has three cysteines, of which Cys20 and Cys23 form an intramolecular disulfide bond within the core of the protein. They are less prone to reduction compared to Cys108, which is located at the protein surface and thus provides a thiol group available for conjugation. Therefore, we covalently conjugated cytochrome c at Cys108 to an azide-modified oligonucleotide using a DBCO maleimide linker (Figure 2A). Under reducing conditions, SDS-PAGE analysis revealed that cytochrome c was conjugated to the oligonucleotide with high efficiency (88%). We also noted additional bands that we attribute to the conjugation of multiple oligonucleotides to a single cytochrome c, possibly through the internal cysteines within the core of the protein. According to our analysis of band intensities by ImageJ, 52% of cytochrome c was conjugated with a single oligonucleotide, 36% was conjugated with two or more oligonucleotides and 12% was left unconjugated (Figure 2B). Staining of the conjugated oligonucleotides with SYBR green further validated these results (Figure A1). Cytochrome c also catalyzes the H_2_O_2_ induced oxidation of a wide range of substrates, including 3,3′,5,5′-tetramethylbenzidine (TMB) [48,49]. Previous studies have shown substantial peroxidase activity of cytochrome c during apoptosis [50]. H_2_O_2_ produced in the mitochondria during apoptosis is used by cytochrome c for the oxidation of cardiolipins residing in the inner mitochondrial membrane [51,52]. This redox modification causes membrane damage and promotes the release of proapoptotic factors into the cytoplasm, which trigger cell death. In the light of these results, we tested the peroxidase activity of cytochrome c with or without ODN conjugates (Figure A2). Our results indicate that modification of cytochrome c through cysteine residue does not impair the crucial peroxidase activity of this protein (*r*_cytc_: 0.018, *r*_cytc-ODN_: 0.012). To confirm the integration of cytochrome c to DNA nanotubes, we covalently labelled cytochrome c first with an Atto488 fluorophore to primary amines (e.g., lysines and arginines), and then conjugated cytochrome c with an azide-modified oligonucleotide to a free cysteine residue. After mixing these oligonucleotides with unconjugated and Alexa Fluor 647-labelled oligonucleotides, the assembled structures were run in 2% agarose gel. Gel analysis revealed that cytochrome c was conjugated to the DNA nanotubes, as the band corresponding to the cytochrome c conjugated DNA nanotubes colocalized in both the 488 nm (Atto) and 647 nm (Alexa Fluor) channels (Figure 2C).

### 3.3. Interaction of DNA Nanotubes with Cancer Cell Membranes

**Figure 3:**
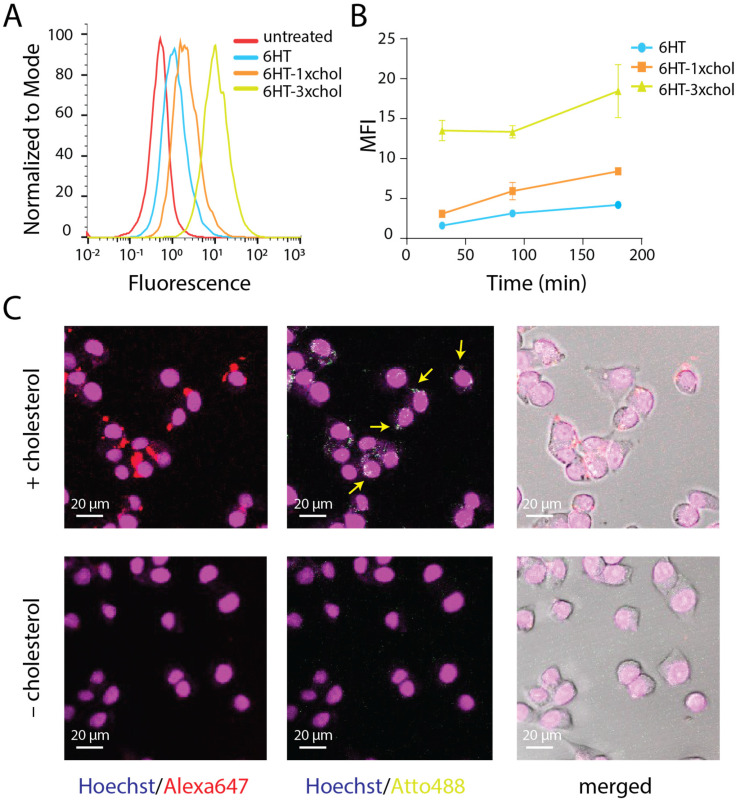
Interaction of DNA nanotubes with HeLa cells. (**A**) FACS analysis of cells treated for 30 min with Alexa Fluor 647-labelled DNA nanotubes. (**B**) Time-dependent binding of DNA nanotubes with varying numbers of cholesterol with HeLa cell membranes. (**C**) Confocal imaging of cells treated with DNA nanotubes labelled with Alexa Fluor 647 and conjugated to Atto488- labelled cytochrome c. Scale bars: 20 µm.

We next addressed the interaction of our designed nanotubes with cancer cells. For this purpose, we chose the cervical cancer cell line HeLa, which is commonly used to study the interaction of DNA nanostructures with cell membranes. We used flow cytometry to quantify the impact of the number of cholesterol anchors on DNA nanotubes, on cell surface binding. For this purpose, cells were incubated with DNA nanotubes (100 nM) for up to 3 h at 37 °C. After 30 min of incubation, the mean fluorescence intensity (MFI) of cells treated with DNA nanotubes containing three cholesterol moieties was 10-fold higher compared to the MFI obtained with unmodified DNA nanotubes, whereas DNA nanotubes carrying 1 cholesterol showed only a 2-fold-higher MFI in comparison to the unmodified nanotubes (Figure 3A). The increase in hydrophobic interaction with the cell membrane or, possibly, an increased aggregation of DNA nanostructure in the solution could both explain the enhanced cell surface binding of the DNA nanotubes modified with three cholesterol moieties. Kinetic analysis showed that the MFI of cells incubated with the different nanotubes increased only minimally upon longer periods of incubation, i.e., 90 min or 3 h, indicating that binding of DNA nanotubes to cell membranes occurred rapidly, mostly within the first 30 min of incubation (Figure 3B). Similar binding kinetics of DNA nanotubes have been reported in recent studies [32,35].

To further analyze cell surface binding and the possible internalization of DNA nanotubes, we performed confocal microscopy imaging. After 30 min of incubation with DNA nanotubes (3× chol) a red fluorescent signal arising from DNA nanotube clusters was clearly observed. The prevalent localization of the fluorescence to a perinuclear region, rather than at the cell periphery or the edges where cell adhesion molecules are normally concentrated (Figure 3C), suggests that these clusters were internalized. The green, fluorescent signal of cytochrome c was also visible around the cell nucleus as highlighted by arrows (Figure 3C). No fluorescent signal was observed in the cells incubated with unmodified DNA nanotubes (0× chol).

**Figure 4:**
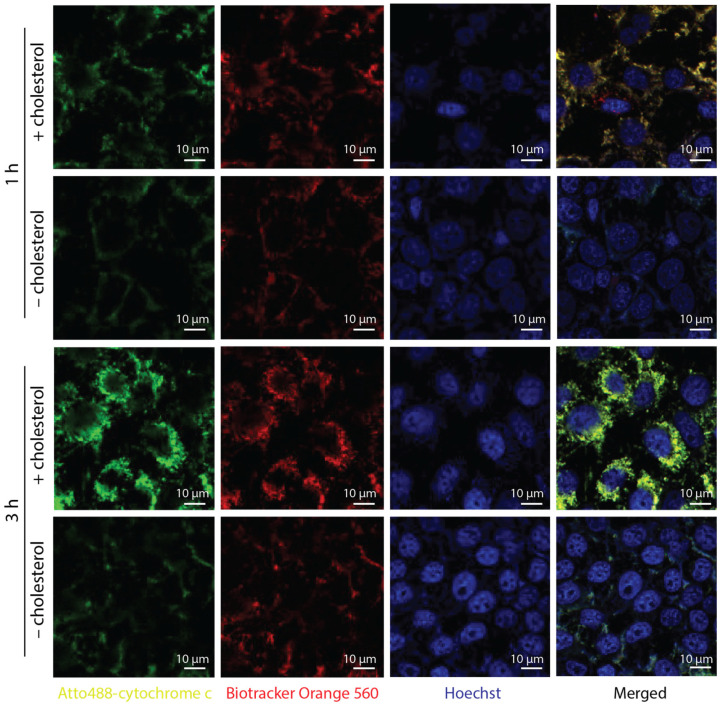
Confocal microscopy analysis of the cellular uptake and internalization of cytochrome c-conjugated DNA nanotubes. Cells treated with cytochrome c-conjugated DNA nanotubes (either having or lacking cholesterol) were imaged after 1 and 3 h of incubation. Cytochrome c was labelled with Atto488 before assembly of DNA nanotubes. Colocalization of nanotubes inside endosomes stained with Biotracker orange 560 lysosome dye. Scale bar: 10 µm.

**Figure A3:**
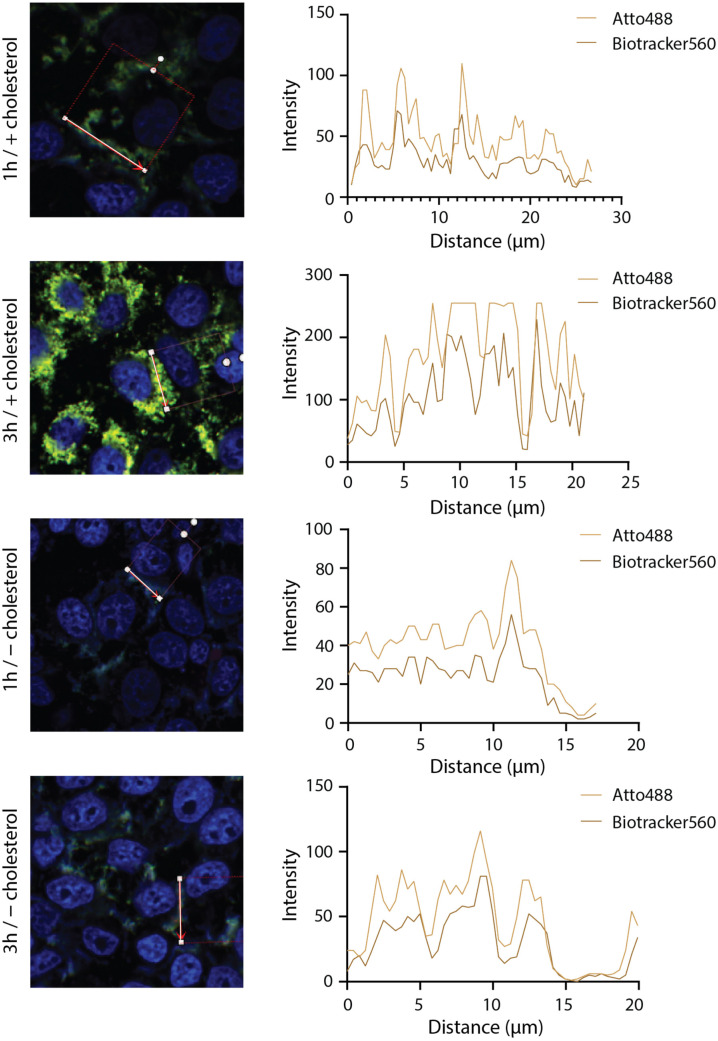
Fluorescent intensity profile analysis of cells incubated with Atto488 labelled cytochrome c (green) and Biotracker560 (red) along the arrowed lines.

**Figure A4:**
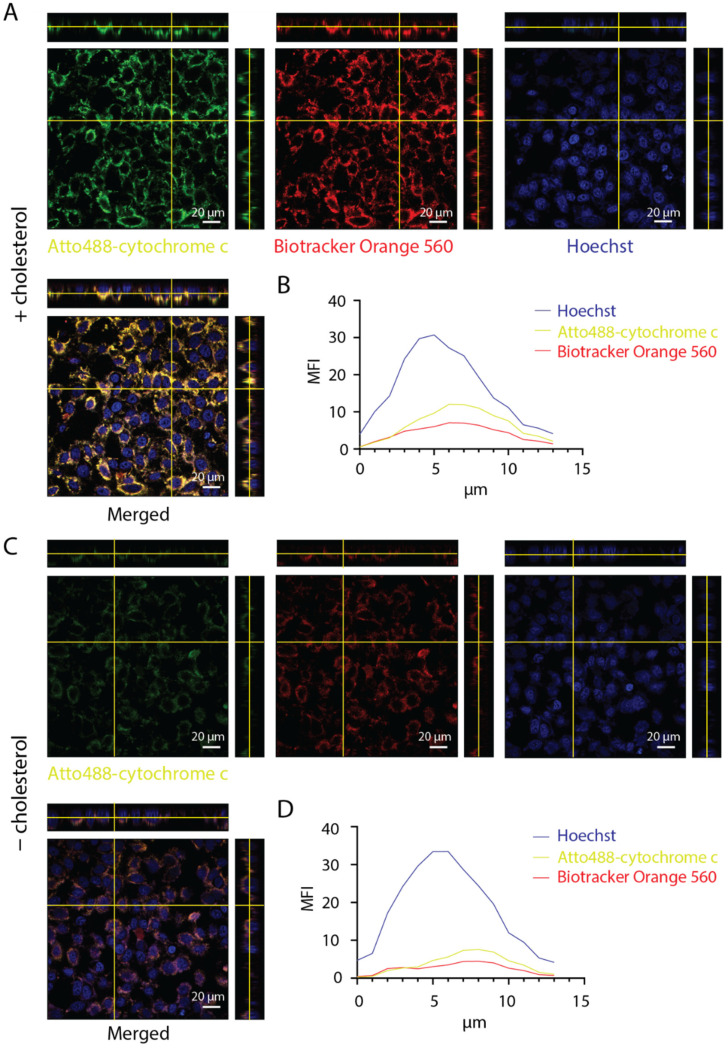
Internalization of cytochrome c-conjugated DNA nanotubes. (**A**) Orthogonal *xz* and *yz* cross sections of cells treated with cholesterol-conjugated DNA nanotubes. (**B**) Fluorescent intensity profile of the respective *z*-stack image. (**C**) Orthogonal *xz* and *yz* cross sections of cells treated with DNA nanotubes without cholesterol molecules. (**D**) Fluorescent intensity profile of the respective *z*-stack image. Scale bar: 20 µm.

To obtain further evidence for the internalization of the nanotube, we incubated cells with cholesterol-modified and unmodified, cytochrome c-conjugated DNA nanotubes for 1 h and 3 h and then with Hoechst and Biotracker 560, a molecule that accumulates in endosomes, for an additional 30 min. We observed that the green, fluorescent signal of the cytochrome c-conjugated DNA nanotubes (Atto488) colocalized with the red Biotracker 560 signal after 1 h of incubation (Figure 4). Fluorescent intensity analysis by Zen software (3.3) indicated that the green signal of cytochrome c and the red signal of Biotracker 560 (endosomes) consistently overlapped across multiple cells (Figure A3). The fluorescent signal of Atto488-labelled cytochrome c was higher when the DNA nanotubes carried cholesterol, consistent with the effect of cholesterol on membrane binding and internalization of the nanotube. With longer incubation times, accumulation of cytochrome c in endosomes increased. However, its localization remained limited to the endosomal/perinuclear compartment. The internalization of DNA nanotubes and the localization of cytochrome c in endosomes was further visualized by *z*-stack imaging (Figure A4). Enhanced fluorescent intensities inside the cells were visible in the *z* direction when DNA nanotubes were modified with cholesterol. From these results, we concluded that cytochrome c was internalized and trapped in the endosomal system but not released into the cytosol. The fluorescence intensity of DNA nanotubes lacking cholesterol only slightly increased after 3 h of incubation but remained at a fraction of the intensity of the DNA nanotube containing cholesterol (Figure 4).

### 3.4. Induction of Cell Death by DNA Nanotubes

**Figure 5:**
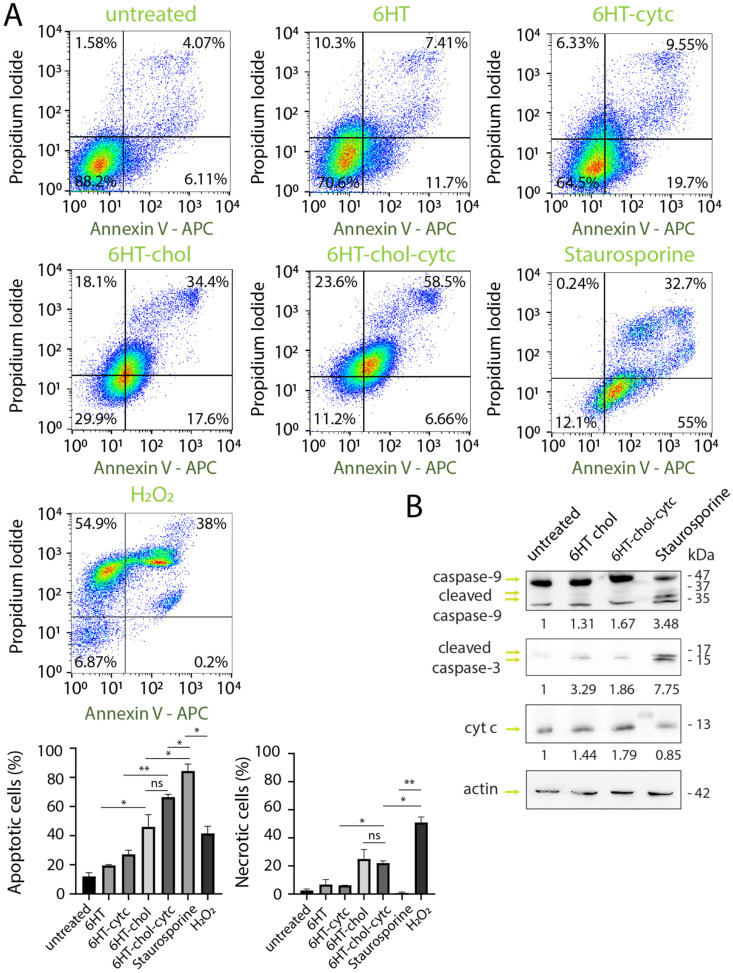
Cholesterol-conjugated DNA nanotubes triggered cell death in HeLa cells. (**A**) Flow cytometry of Annexin V/Propidium Iodide-stained cells after 4 h of treatment with DNA nanotubes showing an increase in apoptotic and necrotic fractions of cell populations treated with the cholesterol-modified DNA nanotubes. The percentage of apoptotic cells for each condition is plotted in the bar graph showing the sum of cells in the right quadrant: Q2 (early apoptotic cells, below) and Q3 (late apoptotic cells, above). The percentage of necrotic cells for each condition is plotted in the bar graph showing the cells in the top left quadrant: Q4 (necrotic cells). *n* = 3. Statistical analysis was performed by an unpaired *t*-test. ns = no statistical difference, * *p* < 0.05, ** *p* < 0.01. (**B**) Western blot analysis to monitor caspase 3, 9 and cytochrome c in HeLa cells 24 h after incubation with DNA nanotubes. Actin was used as loading control, and staurosporine was used as a positive control of apoptosis. Band intensity was determined by normalizing first to actin and then to the untreated control cells.

**Figure A5:**
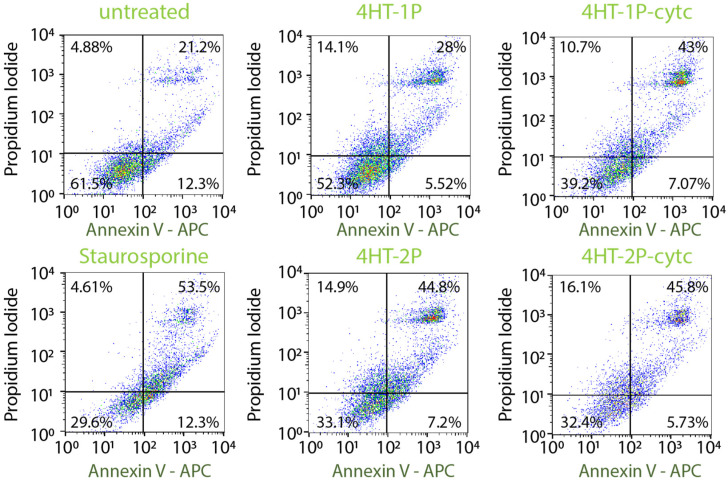
Palmitate-conjugated DNA nanotubes induce cell death in HeLa cells. Flow cytometry analysis of the Annexin V/Propidium Iodide-stained cells, 4 h after treatment with 1 µM palmitate-modified 4-helix DNA nanotubes (4HT). A palmitate-dependent increase of the apoptotic and necrotic fractions in the cell populations was observed.

To evaluate the capacity of the different nanotubes to induce cell death in HeLa cells, we first performed Annexin V/ Propidium Iodide assays after incubation of cells with DNA nanotubes (1µM) for 4 h. This assay detects apoptotic cells through the high-affinity binding of Annexin V protein to membrane phosphatidylserine (PS), which is translocated from the inner to the outer leaflet of the plasma membrane during apoptosis [53]. Propidium Iodide (PI) was used to detect the necrotic/dead cells. It is a membrane-impermeable dye that binds to the nucleic acid only when cells have a disrupted plasma membrane and hence are dead. Flow cytometry analysis revealed that cholesterol-modified DNA nanotubes induced significantly stronger apoptosis than DNA nanotubes without cholesterol (Figure 5A). Compared to untreated cells and plain DNA nanotubes, we observed a significant increase in dying cells (52%, sum of right quarters for cells in the stage of early and late apoptosis, respectively; 18%, top left quarter for cells undergoing necrosis). However, there was no significant increase in the fraction of dying cells when cholesterol-modified DNA nanotubes were conjugated to cytochrome c: 65% of the total population were apoptotic cells, and 23.6% were necrotic cells. As a positive control of apoptosis, we treated cells with 1 µM staurosporine, a pan-protein kinase inhibitor, known to induce apoptosis, and observed that 88% of cells were apoptotic after treatment. As a positive control of necrosis, we treated cells with 3 mM H_2_O_2_ and observed that 54% of cells were necrotic after this treatment. To further understand the effect of the lipid modification on DNA nanotubes on apoptosis, we conjugated the nanotubes with palmitate, a common saturated (16:0) fatty acid. Compared to untreated cells, there was a significant increase in apoptotic cells treated with palmitate-conjugated DNA nanotubes (52%). However, there was again no additive effect when the nanotubes were conjugated with cytochrome c (51.5%) (Figure A5).

**Figure A6:**
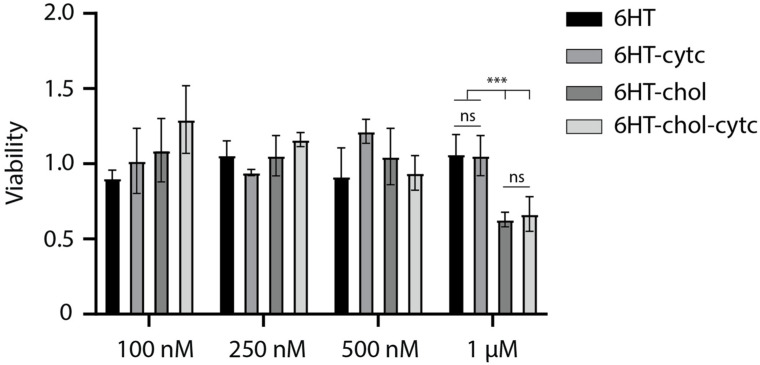
Viability of HeLa cells incubated with different DNA nanotube concentrations. The MTT viability assay of cells demonstrates the viability of cells 24 h after treatment with DNA nanotubes at various concentrations. Absorbance values at 570 nm were normalized to untreated cells. Statistical analysis was performed by an unpaired *t*-test. ns = no statistical difference, *** *p* < 0.005. *n* = 3.

**Figure A7:**
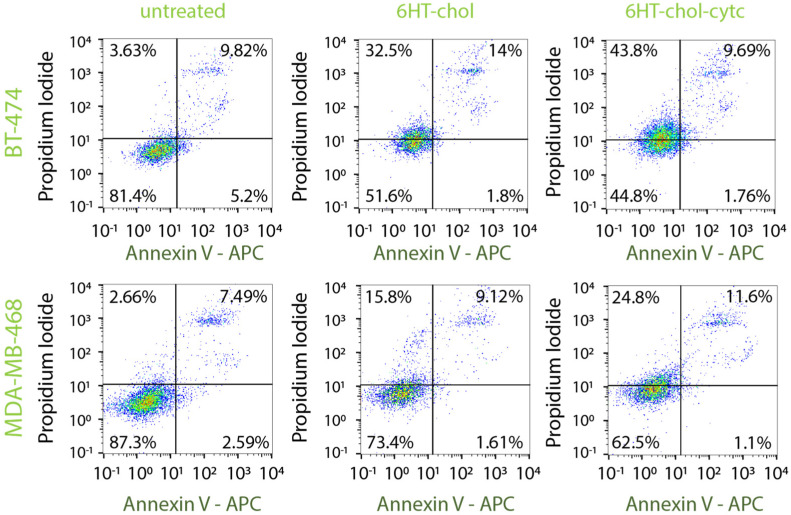
Viability of breast cancer cells (BT-474 and MDA-MB-468) after 24 h of incubation with cholesterol-conjugated DNA nanotubes. Flow cytometry analysis of the Annexin V/Propidium Iodide-stained cells 24 h after incubation with 1 µM cholesterol-modified DNA nanotubes. A cholesterol-dependent increase of the necrotic fractions in the cell populations was observed.

To collect further evidence for the induction of apoptosis, we monitored the activation of caspases after treating cells with cholesterol-modified DNA nanotubes for 24 h by Western blot analysis (Figure 5B). Cleaved (active) caspase 3 and cleaved caspase 9 levels were increased compared to untreated cells. However, they remained lower compared to cells treated with staurosporine for 4 h. Cytochrome c levels were slightly increased when cells were incubated with cytochrome c-conjugated DNA nanotubes, consistent with a delivery of cytochrome c. However, there was no apparent increase in the levels of cleaved caspases due to the uptake of DNA nanotubes containing cytochrome c. Furthermore, cell viability assay also confirmed a cholesterol-dependent cytotoxicity when cells were treated with 1 µM DNA nanotubes (Figure A6). Next, we treated other cancer cells (BT-474 & MDA-MB-468) with cholesterol-modified DNA nanotubes and again observed an increase in the necrotic fractions of the cell populations, further confirming the cytotoxicity of cholesterol-conjugated DNA nanotubes (Figure A7).

**Figure 6:**
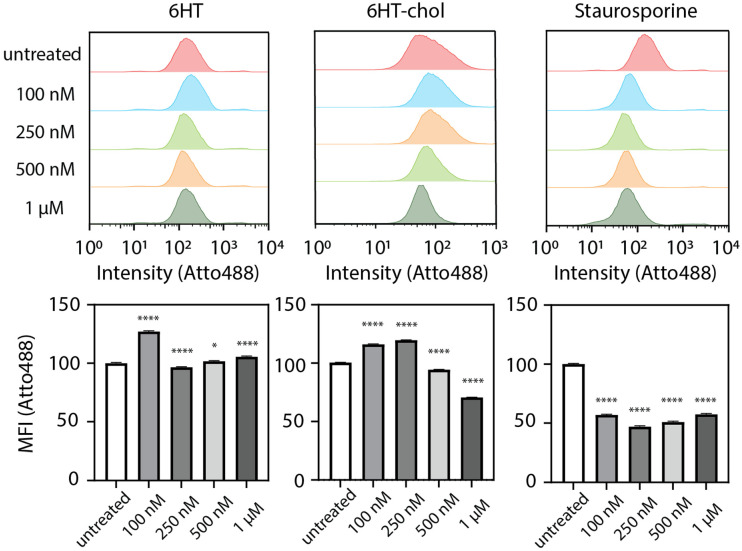
Atto488 efflux upon treatment of HeLa cells with DNA nanotubes. Flow cytometry analysis was performed 3 h after incubation of cells with DNA nanotubes or the apoptosis inducer, staurosporine. The bar graphs below show the mean fluorescence intensity (MFI) normalized to untreated cells. Statistical analysis was performed by an unpaired *t*-test. * *p* < 0.05, **** *p* < 0.0001. *n* = 3.

During apoptosis, cells lose their membrane stability, resulting in swelling and blebbing, but also lose their membrane integrity due to membrane rupture [54]. Recent studies have demonstrated that lipid modified DNA nanopores can penetrate the lipid membranes and allow the transport of cytotoxic drugs across membranes [28,35]. This penetration of the DNA nanopore itself could be cytotoxic [55]. To further validate the possible effects on membrane integrity and leakage caused by cholesterol-modified DNA nanotubes, we preincubated cells with Atto488 dye for 2 h and then treated them with either cholesterol-modified or unmodified DNA nanotubes for an additional 3 h. We observed an efflux of Atto488 dye in cells incubated with cholesterol modified DNA nanotubes at the concentrations of 500 nM and 1 µM, but not with unmodified ones (Figure 6). On the other hand, cells treated with the apoptosis inducer, staurosporine, revealed an Atto488 dye efflux at all concentrations tested (100 nM–1 µM). It is apparent that staurosporine caused membrane damage at lower concentrations compared to the cholesterol-modified DNA nanotubes. However, treatment of cells with lower concentrations of cholesterol-modified DNA nanotubes cause an opposite effect: an increase in fluorescence intensity. This could be explained by variations in autofluorescence of cells. It is well known that cellular autofluorescence is prone to changes in response to their metabolic state. A recent study in bacteria showed an increase in green autofluorescence (excitation at 488 nm with bandpass filters of 530/30) in cells stressed with ampicillin, owing to changes in flavins, including flavin mononucleotide (FMN), flavin adenine dinucleotide (FAD) and riboflavin [56]. This elevation was attributed to the activation of an adaptive response to combat stress. It seems that even though the viability of cells and their membrane integrity is not significantly affected after treatment with the cholesterol-modified nanotubes at 100 and 250 nM concentrations (Figure A6), their autofluorescence nevertheless appears altered.

**Figure A8:**
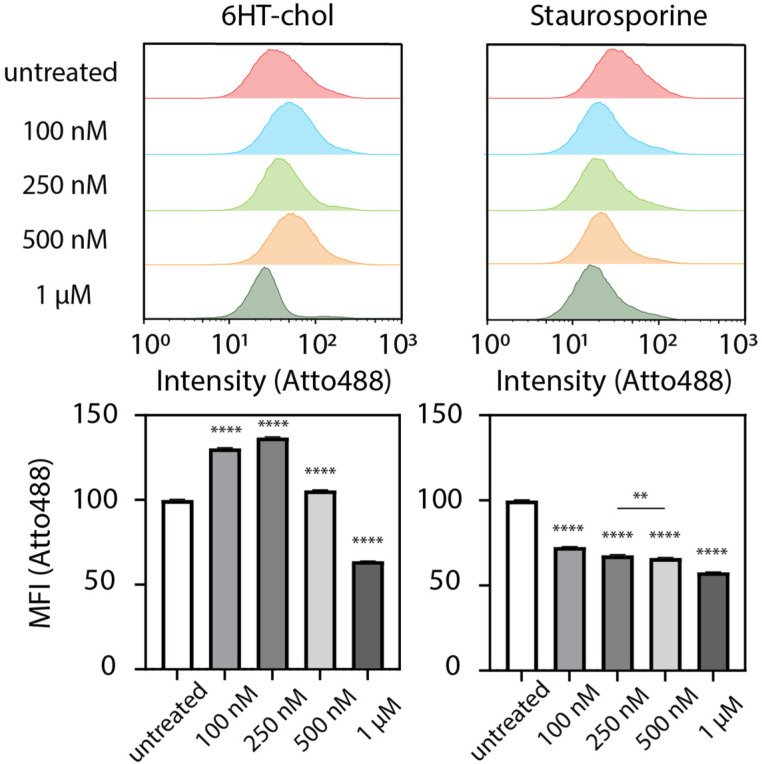
Atto488 efflux upon treatment of HeLa cells with cholesterol-modified DNA nanotubes or staurosporine in the presence of the pan-caspase inhibitor, Z-VAD-FMK. Flow cytometry analysis of cells was performed after 3 h of incubation with DNA nanotubes or the apoptosis inducer, staurosporine, at a concentration range of 100 nM to 1 µM. The bar graphs below show the mean fluorescence intensity (MFI) normalized to untreated cells. Statistical analysis was performed by an unpaired *t*-test. ** *p* < 0.005, **** *p* < 0.0001. *n* = 3.

**Figure A9:**
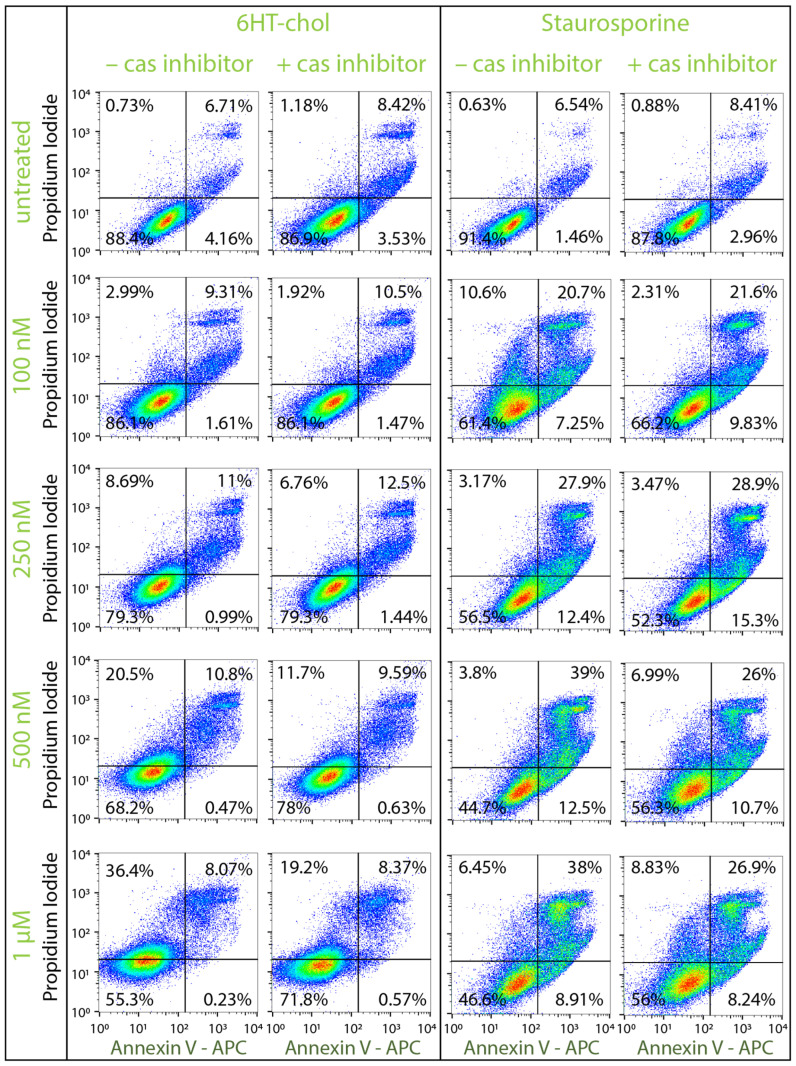
Flow cytometry of the Annexin V/Propidium Iodide-stained cells after 3 h of treatment with cholesterol modified DNA nanotubes (left panel) and the apoptosis inducer, staurosporine (right panel), at a concentration range of 100 nM to 1 µM in the absence or presence of the pan-caspase inhibitor, Z-VAD-FMK (400 µM), as indicated.

To further investigate the underlying mechanism of cell death, we treated cells with the pan-caspase inhibitor, Z-VAD-FMK, prior to the incubation of cells with DNA nanotubes or staurosporine. Flow cytometry analysis revealed that Atto488 dye-efflux was not affected when cells were pretreated with the caspase inhibitor (Figure A8). Moreover, the Annexin V/Propidium Iodide assay indicated a slight decrease in the apoptotic and necrotic cell fractions upon treatment of cells with the cholesterol-modified DNA nanotubes or staurosporine (especially at 500 nM and 1 µM concentrations) in the presence of the pan-caspase inhibitor (Figure A9). This indicates that the pretreatment with the pan-caspase inhibitor only partially prevents cell death, implying the involvement of additional, caspase-independent mechanisms of cell death. It was previously shown that staurosporine can induce apoptosis through both caspase-dependent and -independent mechanisms in leukemia cell lines [57], and treatment with the pan-caspase inhibitor could reduce the rate of cell death by up to 40%. Overall, these experiments reveal caspases cleavage upon treatment with cholesterol-modified DNA nanotubes and staurosporine, but caspase inhibition does not appear to be sufficient to fully prevent the induction of cell death.

## 4. Discussion and Conclusions

In this study, we developed cholesterol-modified DNA nanotubes that bind to and are taken up by HeLa cervical cancer cells, and induce cell death associated with caspase activation and increased membrane permeability. However, irreversible caspase inhibition only partially prevented cell death, consistent with a combined form of apoptotic and necrotic cell death. Our results show that induction of cell death requires the modification of the DNA nanotubes with lipids, either cholesterol or palmitic acid, but not cytochrome c. The hydrophobic part of the structure decorated with lipid molecules was designed to span the lipid bilayer so that the conjugated cytochrome c could reach the cytoplasm upon membrane insertion. Using flow cytometry and confocal microscopy, we observed that lipid-modified DNA nanotubes bound the cell surface efficiently, and that they were taken up by the cells to localize to a perinuclear/endosomal compartment. The lipid-dependent cytotoxicity of nanotubes is likely due to their efficient surface targeting and uptake compared to unmodified nanotubes. Their cytotoxicity is possibly due to their ability to affect the integrity of the plasma membrane (Figure 6). DNA nanotubes are known to have pore-like properties and can thus disrupt the membrane potential. Alternatively, they might exhibit cytotoxic effects once they are taken up by the cells and might potentially disturb trafficking through the endosomal system.

The membrane potential is essential for cell viability. In nature, there are various examples of pore-forming peptides that target the cell membrane and thereby induce cell death. These include the antibacterial channel-forming peptides, such as alamethicin, produced by the fungus *Trichoderma viride* and ceratotoxin A, produced by the medfly *Ceratitis capitata* [58,59]. Pore-forming toxins made of proteins such as the cholesterol-dependent cytolysin can also be secreted by gram-positive bacteria and induce apoptosis upon pore formation on the target cell membrane [60]. However, from our results, we cannot distinguish whether the cytotoxicity of the cholesterol-conjugated DNA nanotubes is due to their possible pore-forming activity at the plasma membrane, or whether they become toxic only once they are taken up by the cells, and possibly affect trafficking through the endosomal system. Previously, it has been demonstrated that a DNA nanopore with a hydrophobic belt, composed of ethyl phosphorothioate, induced cytotoxicity on HeLa cells within 1 h at a concentration of 100 nM [55]. In another study, 50 nM phosphorothioate-modified DNA nanopore induced 20% cytotoxicity after 40 min of incubation. However, no cytotoxicity was observed when cells were treated at 4 °C. In other words, the phosphorothioate-modified DNA nanopore blocked endocytosis. Thereby, the DNA nanopores were retained at the cell surface [35]. In our experiments, we observed cholesterol-dependent cytotoxicity when cells were incubated with DNA nanotubes within a range of 500 nM to 1 µM (Figure 5, Figure A6 and Figure A9). On the other hand, we could not observe the endosomal escape of cytochrome c and cytochrome c-triggered apoptosis at 1 µM of the DNA nanotubes, a concentration much lower compared to other studies, where cancer cells were treated with 10–50 µM cytochrome c containing nanoparticles [42,43]. To increase the capacity of cytochrome c to escape from the acidic environment of the endosomal compartment, other disulfide-based linkers, such as Sulfo-LC-SPDP (sulfosuccinimidyl 6-[3′-(2-pyridyldithio)propionamido]hexanoate) and DBCO-S-S-NHS ester, which could be reduced at low pH, might be tested. Furthermore, more sophisticated designs to puncture lipid membranes can be tested in which cytochrome c is conjugated to the long inner helix and cholesterol moieties are conjugated to the surrounding shorter outer helices. This would allow more efficient penetration into the membrane, as such structures would be more similar to the natural pore-forming protein α-hemolysin [25,61]. Moreover, other DNA-based polyhedral designs, such as tetrahedrons [62] or icosahedrons [63], could be an alternative strategy to encapsulate cytochrome c within their inner void.

The cytotoxic effect of DNA nanotubes observed in this study could be applicable to other cancer cell types by combining the cholesterol-mediated cellular binding of DNA nanotubes with antibody/nanobody conjugations, peptides or DNA/RNA aptamers to specifically target cancer antigens. Lipidomic analyses have revealed significant lipid alterations in cancer cells in terms of both lipid class and their molecular species composition. One remarkable change was observed in the membrane phospholipids, which are crucial for adaptation to carcinogenic processes by orchestrating membrane fluidity and signal transduction [64]. The enrichment of saturated phospholipids (particularly in phosphatidylcholine, phosphatidylethanolamine) is regarded as a signature of cancer cells [65], and enriched saturated phospholipids are well known for their contribution to protect against lipid peroxidation [66]. These saturated lipid species also promote interactions with cholesterol [67]. We expect that our cholesterol-modified DNA nanotubes display a higher affinity to cancer cells and hence increase the efficiency of their treatment. In conclusion, the lipid-modified DNA nanotube-based structures designed here provide a promising tool for targeted cell therapies through their increased interactions with cellular membranes, particularly those of malignant cells.

## Data Availability

The raw data supporting the conclusions of this article will be made available by the authors upon request.

## Appendix A. Supporting Figures & Tables

**Table A1:** Sequences of oligonucleotides used in 6-helix DNA nanotube assembly. All conjugated oligonucleotides were purified using HPLC.

Oligonucleotide	Sequence	Modification
U1R1	AAAAACTTACTGAGGATATTGCCTGAAGCTGTACCGTTTTAGGGGAAA	5′ azide
U1R2	ACGTACCTGAACTCCAGACTCGGGGCGAAAAAAAAGTCTTAGTACCGC	3′ chol
U2R1	CCCCTAAAACGGTACAGCTTCGGTACGTGCGGTACTAAGACTGGGGCGAATAGACAGGCTCCCCTCTCACTCGCTAGGAGGCAA	
U3R1	AAATTGCCTCCTAGCGAGTGAGAGGTTTCCCGCATATTAACGCCTAAA	
U3R2	CAGCGTCGGGAGCCTGTCTATTCGCCCCAAAAAATACCGTGGTTGAGT	3′ chol
U4R1	AGGCGTTAATATGCGGGAAACGACGCTGACTCAACCACGGTACGTTAGATGCCTCGCTGTACTAATAGTTGTCGACAGATCGTC	
U5R1	AAAGACGATCTGTCGACAACTATTGGTCGGATCTGAGTCGACCAAAAA	5′ Atto647
U5R2	ATGGAGCAGTACAGCGAGGCATCTAACGAAAAAAGCTCTTTGAGTATC	3′ chol
U6R1	TTGGTCGACTCAGATCCGACCGCTCCATGATACTCAAAGAGCTCGCCCCGAGTCTGGAGTTCAAGGCAATATCCTCAGTAAGTT	

**Table A2:** Antibodies used in the Western blot analysis.

Antibody	Source	Dilution
Anti-caspase 9	Cell Signaling, Danvers, MA, USA (#9502)—rabbit	1:1000
Anti-cleaved caspase 3 (Asp175)	Cell Signaling, Danvers, MA, USA (#9661)—rabbit	1:1000
Anti-cytochrome c	Biolegend, San Diego, CA, USA (#612503)—mouse	1:500
β-actin	Sigma Aldrich, Buchs, Switzerland (#A3853)—mouse	1:10,000
Goat-anti mouse Ig/HRP	Dako, Glostrup, Denmark (#P0447)	1:10,000
Goat-anti rabbit Ig/HRP	Dako, Glostrup, Denmark (#P0448)	1:10,000
